# Genome-Wide Association Study Using Individual Single-Nucleotide Polymorphisms and Haplotypes for Erythrocyte Traits in Alpine Merino Sheep

**DOI:** 10.3389/fgene.2020.00848

**Published:** 2020-07-31

**Authors:** Shaohua Zhu, Tingting Guo, Hongchang Zhao, Guoyan Qiao, Mei Han, Jianbin Liu, Chao Yuan, Tianxiang Wang, Fanwen Li, Yaojing Yue, Bohui Yang

**Affiliations:** ^1^Animal Science Department, Lanzhou Institute of Husbandry and Pharmaceutical Sciences, Chinese Academy of Agricultural Sciences, Lanzhou, China; ^2^Sheep Breeding Engineering Technology Center, Chinese Academy of Agricultural Sciences, Lanzhou, China; ^3^Gansu Provincial Sheep Breeding Technology Extension Station, Sunan, China

**Keywords:** genome-wide association study, single-nucleotide polymorphism, haplotype, high-altitude hypoxia adaptation, erythrocyte traits, alpine merino sheep

## Abstract

Adaptation to high-altitude hypoxia is essential for domestic animals, such as yak, Tibetan chicken, and Tibetan sheep, living on high plateaus, as it ensures efficient oxygen absorption and utilization. Red blood cells are the primary medium for transporting oxygen in the blood. However, little is known about the genetic mechanism of erythrocyte traits. Genome-wide association studies (GWASs) based on single markers or haplotypes have identified potential mechanisms for genetic variation and quantitative traits. To identify loci associated with erythrocyte traits, we performed a GWAS based on the method of the single marker and haplotype in 498 Alpine Merino sheep for six erythrocyte traits: red blood cell count (RBC), hemoglobin (HGB), hematocrit (HCT), mean corpuscular hemoglobin (MCH), mean corpuscular hemoglobin concentration (MCHC), and RBC volume distribution width coefficient of variation (RWD_CV). Forty-two significant single-nucleotide polymorphisms (SNPs) associated with the six erythrocyte traits were detected by means of a single-marker GWAS, and 34 significant haplotypes associated with five erythrocyte traits were detected by means of haplotype analysis. We identified six genes (*DHCR24, SPATA9, FLI1, PLCB1, EFNB2*, and *SH2B3*) as potential genes of interest via gene function annotations, location, and expression variation. In particular, *FLI1* and *PLCB1* were associated with hematopoiesis and erythropoiesis, respectively. These results provide a theoretical basis for analyzing erythrocyte traits and high-altitude hypoxia adaptation in Alpine Merino sheep and will be a useful reference for future studies of plateau-dwelling livestock.

## Introduction

The sheep (*Ovis aries*) was one of the first animals to be domesticated by humans (Zeder, 2008; Wang et al., 2014). Sheep provide a variety of resources, including wool, mutton, and milk, and thus play an indispensable role in the global agricultural economy. Merino and Merino-derived sheep breeds are widely distributed around the world; they represent vital genetic resources, both historically and economically, and have become the basis for breeding new varieties (Ciani et al., 2015). The Alpine Merino sheep is a new breed of Merino, developed by crossing Australian Merino sheep with Tibetan sheep, and lives in areas of the Qinghai-Tibet Plateau (average elevation >4500 m above sea level) all year round. In addition to the quality of wool and meat they provide, they have the advantages of resistance to rough feeding and adaptation to high-altitude hypoxia. The latter adaptation refers to a process of morphological, physiological, and biochemical changes that occur in humans or animals that have survived and evolved in the hypoxic environment of the plateau for generations, and are thus well adapted to that environment. This adaptation is the result of the survival and evolution of populations (including humans and animals) in the hypoxic environment, and it can be stably inherited (Peng et al., 2017). It is an efficient way for the body to exist in a low-oxygen environment, allowing populations to use the lower level of oxygen (60% of that at sea level) on the plateau to complete normal physiological functions and survive in the harsh environment.

Hemocytes (blood cells) have a vital function in conferring resistance to diseases and in the transport of oxygen (Müller and Brem, 1991). They include three main cell types: erythrocytes (red blood cells, RBCs), leukocytes (white blood cells), and thrombocytes (platelets) (Kickler, 2005). Of these three components, RBCs are crucial for the transport of oxygen and the removal of respiratory byproducts in organisms. To be utilized by the body, oxygen from the external environment needs to be transported and exchanged via the lungs, transported through the blood, and exchanged between the blood and the various tissues in the body. The viscosity of blood and the deformability of RBCs play a crucial role in transporting and exchanging oxygen; several studies have shown that the viscosity of blood depends on existing shear forces, RBC aggregation, the hematocrit (HCT, vol% of RBCs in blood), and the biomechanical properties of RBCs (Chien, 1987; Tripette et al., 2009). Other erythrocyte traits, including mean corpuscular hemoglobin (MCH) and mean corpuscular hemoglobin concentration (MCHC), are associated with impaired deformability of RBCs (von tempelhoff et al., 2015). In addition, the oxygen-carrying capacity of the blood is affected by the HCT and HGB content (Eklom and Lill, 2006). Thus, in the processes noted above, erythrocytes have essential functions in oxygen storage, binding, transportation, and release (Balasubramanian et al., 2009).

The genome-wide association study (GWAS) was originally developed as a means to screen genetic loci associated with complex diseases, and it is gradually becoming an effective tool for locating genetic sites associated with quantitative traits (Lockett and Holloway, 2013; Verstockt et al., 2018). In recent years, GWAS and quantitative trait loci (QTL) mapping have been widely used in research in animals, including pig (Wang et al., 2017), cattle (McDaneld et al., 2014), sheep (Kominakis et al., 2017), horse (Dupuis et al., 2011), and chicken (Zhang et al., 2013), as well as in humans (Davenport et al., 2015). Moreover, many traits have been evaluated in these studies, such as wool (Wang et al., 2014) and milk production (Fang et al., 2017), growth (Crispim et al., 2015), reproduction (Hawken et al., 2011), immune response (Go et al., 2012), and also high-altitude hypoxia adaptation (Hendrickson, 2013).

Several previous studies have reported gene function screening and QTL mapping results for high-altitude hypoxia adaptation in humans, pig, yak, Tibetan chicken, and Tibetan sheep (Zhang et al., 2007; Qiu et al., 2012; Dunn et al., 2013; Wei et al., 2016). Simonson et al. (2010) found three genes, *EPAS1* (endothelial PAS domain protein 1), *EGLN1* (egl-9 family hypoxia inducible factor 1), and *PPARA* (peroxisome proliferator activated receptor alpha), associated with HGB concentration by whole-genome scan. Favier et al. (2001) found that most of the factors or enzyme changes caused by hypoxia are regulated by *EPAS1* and noted that the latter was a crucial transcription factor for stress hypoxia. Zhang et al. (2007) measured hematological characteristics in Tibetan chickens at different ages and found that the chickens retained a high level of RBCs and a stable level of hematocrit as they aged. Wei et al. (2016) studied the relationship between blood-related phenotypes and *EPAS1* genotypes in Tibetan sheep by genome-wide analysis and showed that *EPAS1* was associated with increased MCHC and mean corpuscular volume. Haase et al. found that, under low-oxygen conditions, the body can stimulate the synthesis and release of erythropoietin through cellular oxygen-sensing pathways such as HIF-1X, thus promoting hemoglobin production and erythrocyte proliferation to adapt to a harsh environment (Haase, 2013). Yang et al. (2016) found that *HMOX2* (also known as *HO-2*, heme oxygenase 2) as a modified gene could regulate the metabolism of downstream hemoglobin in the hypoxic metabolic pathway, indicating a mechanism for genetic adaptation of the Tibetan population to low-altitude hypoxia. Zou et al. (2008) identified 101 QTLs affecting RBCs traits on wild boar (*Sus scrofa*) chromosomes 1, 2, 3, 4, 5, 6, 7, 8, 9, 10, and 13. However, as mentioned in the above study, some of the candidate genes detected in the results were consistent, but some new candidate genes have also been discovered. This may reflect the complex genetic mechanisms of blood cells and the differences between species.

To the best of our knowledge, few GWASs have been conducted in sheep than in humans and pigs because of the limited information available for the sheep genome. Moreover, there is no specific parameter defined for high-altitude hypoxia adaptation, and it can be reflected by related characteristics such as erythrocyte traits, cardiopulmonary function, and oxygen circulation in cells and tissues. Therefore, studies of high-altitude hypoxia adaptation in sheep are rarely reported. The Alpine Merino sheep involved in this study had a mixture of Australian Merino sheep and Tibetan sheep ancestry, they quickly adapting to the Qinghai-Tibet Plateau and surviving in high altitude and hypoxia adaption, with the consequence that they present an ideal subject for experiment in adaptation to high-altitude hypoxia. The main objectives of the present study were to detect significant SNPs for erythrocyte traits in Alpine Merino sheep by using the custom Affymetrix HD 630K microarrays and to explore the potential genes associated with high-altitude hypoxia adaptation.

## Materials and Methods

### Ethics Statement

All the animal work involved in this study was conducted according to the guidelines for the care and use of laboratory animals promulgated by the State Council of the China. This research was approved by the Animal Management and Ethics Committee of Lanzhou Institute of Animal Husbandry and Veterinary Medicine, Chinese Academy of Agricultural Sciences (license number: 2019-008).

### Animal Samples and Phenotypes

The sheep population used in this study was selected from Huangcheng pasture in Gansu Province, China, part of the Gansu Provincial Sheep Breeding Technology Extension Station. The station has a strictly standardized breeding and management system, which ensured the health of each individual sheep involved in the experiment. The flock consisted of 501 male Alpine Merino sheep, aged 18–24 months. Blood samples (5 mL) from each of the 501 sheep were collected from the jugular vein and transferred immediately to a vacuum blood collection tube (Yuli Medical Equipment Company Ltd., Jiangsu Province, China).

A standard set of erythrocyte data was recorded using a H-100iv diff whole blood analyzer (Sysmex, Kobe, Japan) within 24 h of specimen collection at Lanzhou Institute of Animal Husbandry and Veterinary Medicine, Chinese Academy of Agricultural Sciences. The remainder of each blood sample was stored at −20°C after the hematological data were recorded. Six erythrocyte traits closely associated with high-altitude hypoxia adaptation were then selected: red blood cell count (RBC), hemoglobin (HGB), hematocrit (HCT), mean corpuscular hemoglobin (MCH), mean corpuscular hemoglobin concentration (MCHC), and red blood cell volume distribution width coefficient of variation (RWD_CV). Then, the phenotype data were adjusted through remove the extreme values that exceeded three times the standard deviation before conducting the association study.

### Genotyping and Quality Control

In total, 501 Alpine Merino sheep were genotyped using the custom Affymetrix HD 630K microarrays, and the genotyping platform adopted in this study was based on the Array Plate Processing Workflow for the Thermo Fisher Scientific (Affymetrix) GeneTitan System (Santa Clara, CA, United States). Before statistical analysis, SNPs were preprocessed using PLINK v1.9b4 (Purcell et al., 2007). SNPs were selected based on minor allele frequency >0.01, proportion of missing genotypes <0.05, and a Hardy–Weinberg equilibrium *P*-value > 10e−6. Samples with more than 10% missing genotypes were eliminated. According to the definition given by Gabriel, if the 95% confidence upper boundary on one side of D’ was lower than 0.98 (that is, there is no historical reorganization), and the lower boundary was higher than 0.7, then the pair was considered to be a “strong LD.” If the D’ of a pair of SNPs was lower than 0.7, the next haplotype block was initiated (Gabriel et al., 2002). The reference thresholds and division criteria for tightly linked haplotype internal SNPs were established based on the recommendations of the PLINK software user manual (Purcell et al., 2007). A total of 498 individuals with 439,398 autosomal SNPs and 89,676 haplotypes blocks with 325,062 haplotypes passed filters and quality controls, and these standard-compliant samples were collated for subsequent statistical analysis.

### Statistical Analyses

The method of Bonferroni calibrated multiple tests (Yang et al., 2006) was used to determine significance thresholds. The threshold of the genome-wide significance level of each SNP and haplotype was 0.05, and the threshold of the suggestive or chromosomal significance level of each effective SNP site and haplotype was 1. After calibration of the significance thresholds, the corresponding thresholds for the SNP sites were set as 1.14e−7 (0.05/439,398) and 2.28e-6 (1/439,398); similarly, the corresponding thresholds for haplotypes were set as 1.54e-7 (0.05/325,062) and 3.08e-6 (1/325,062). Based on the whole genome sequence information, we used TASSEL 5.2.43 software (Bradbury et al., 2007) to perform principal component analysis on all the individuals involved in the study, then constructed and drew the principal component analysis plot. In addition, the R package “CMplot” was used to create Manhattan plots and quantile-quantile (Q-Q) plots for each GWAS result^1^

### Studies of Single-Marker Association

The associated data for each SNP through regression analysis based on the generalized linear model (GLM) given below by Eq. (1) (Breslow and Clayton, 1993; McCulloch and Neuhaus, 2015):

(1) $$y=X\,b_{i}+Q\,v+e$$

where *y* is the corrected phenotype, *b*_*i*_ is the regression coefficient of the phenotype on the SNPs, *X* represents the vector of the corresponding SNP indicators, *v* represents the effect of population structure, *Q* is the corresponding principal components matrix, *e* is the vector of residual errors with $e∼N\,\left(0,I\,\sigma_{e}^{2}\right)$, *I* is the identity matrix, and $\sigma_{e}^{2}$ is the variance of *e*. The single-marker analysis was conducted using TASSEL 5.2.43 software (Bradbury et al., 2007).

### Studies of Haplotype-Based Association

Similarly to the single-marker analysis, GLM was adopted to perform the regression analysis in the haplotype-based association studies; *y*, *v*, *Q*, and *e* were as defined previously, *b*_*i*_ represents the regression coefficient of the phenotype on the haplotype blocks, and *X* represents the vector of the corresponding haplotype indicators. Furthermore, the standard expectation maximization (EM) algorithm was used to detect individual haplotype blocks and haplotype frequencies. Specifically, EM iterations interact between performing the expectation step (ES) and establishing the maximized step (MS). The ES and MS establish an equation for the expected value of the log-likelihood, which is evaluated using the estimate of the haplotype and frequencies (Excoffier and Slatkin, 1995). According to recommendations of the PLINK software user manual (Purcell et al., 2007), we established the reference thresholds and division criteria for tightly connected haplotype internal SNPs. The haplotype-based analysis was also conducted using the TASSEL 5.2.43 software (Bradbury et al., 2007).

## Results

### Phenotypic Statistics and Genotypic Characteristics

**FIGURE 1:**
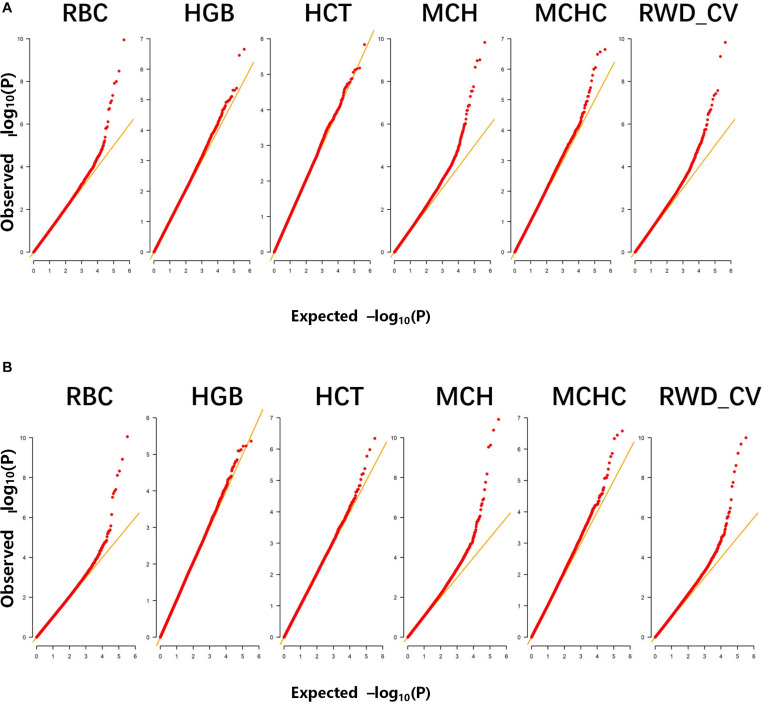
Quantile-quantile (Q–Q) plots of genome-wide association studies (GWASs) for six erythrocyte traits for: **(A)** single-marker analysis and **(B)** haplotype analysis. The six traits were: red blood cell count (RBC), hemoglobin (HGB), hematocrit (HCT), mean corpuscular hemoglobin (MCH), mean corpuscular hemoglobin concentration (MCHC), and RBC volume distribution width coefficient of variation (RWD_CV).

A total of 498 Alpine Merino sheep were used in this study, all from Huangcheng pasture in Gansu Province, China. Six phenotypic observations related to erythrocyte traits were measured and recorded: RBC, HGB, HCT, MCH, MCHC, and RWD_CV. Descriptive statistics of the phenotypic measurements (Table 1) include the mean, standard deviation, and coefficient of variation. The Q-Q plots for the test statistics from the GLM are shown in Figures 1A,B and indicate that there was no overall systemic bias in the analysis based on single-marker or haplotype analysis. In addition, the structure of this population is drawn based on the top three eigenvectors using principal component 1 (PC1), 2 (PC2), and 3 (PC3), the PCA plot (Supplementary Figure S1) suggests that some of the individuals have population stratification, therefore, the population stratification should be corrected, it could effectively control the group stratification. As shown in Eq. (1), *v* and *Q* represent the influence of the population structure and the corresponding principal component matrix, respectively.

**TABLE 1:** Descriptive statistics of phenotype values of erythrocyte traits.

**Trait**	**Abbreviation**	**Mean ± SD**	**CV^1^ (%)**	**Number**
Red blood cell count (10^12^/L)	RBC	7.66 ± 1.69	22.08	494
Hemoglobin (g/L)	HGB	98.60 ± 11.64	11.80	489
Hematocrit (%)	HCT	0.27 ± 0.08	29.84	496
Mean corpuscular hemoglobin (pg)	MCH	13.24 ± 2.64	19.93	492
Mean corpuscular hemoglobin concentration (g/L)	MCHC	377.47 ± 103.77	27.49	488
RBC volume distribution width coefficient of variation	RWD_CV	0.39 ± 0.07	19.23	498

*^1^ CV, coefficient of variation.*

### Significant SNPs

**FIGURE 2:**
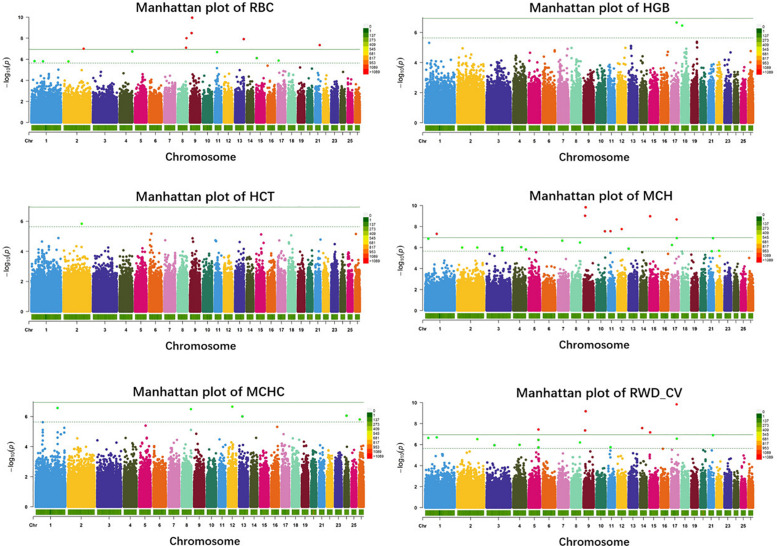
Manhattan plots of single-marker analysis for six erythrocyte traits. Chromosomes 1 to 26 are shown in different colors; the horizontal dotted green line and solid line indicate the suggestive and genome-wide significance level, respectively. Single-nucleotide polymorphisms (SNPs) surpassing the genome-wide threshold are highlighted in red, and SNPs reaching the suggestive threshold are highlighted in green. The strips below each chromosome indicate the density of the markers. The six traits were: red blood cell count (RBC), hemoglobin (HGB), hematocrit (HCT), mean corpuscular hemoglobin (MCH), mean corpuscular hemoglobin concentration (MCHC), and RBC volume distribution width coefficient of variation (RWD_CV).

In single-marker analysis, we detected 42 significant SNPs, including 27 suggested and 15 genome-wide significant SNPs. These associated with six erythrocyte traits (Supplementary Table S1): 14 for RBC, 2 for HGB, 1 for HCT, 24 for MCH, 6 for MCHC, and 20 for RWD_CV, some of which were pleiotropic (12_39476100 were associated with both MCH and MCHC and 8_82239902 were associated with both RBC and RWD_CV). These SNPs were distributed on 20 autosomes, including Oar1, 2, 3, 4, 5, 7, 8, 9,10, 11, 12, 13, 14, 15, 17, 18, 21, 22, 24, and 26 (Figure 2). Of these 42 SNPs, 21 were located within genes and the others were located from 1,461 to 635,535 base pairs (bp) away from the nearest genes (Supplementary Table S1). The most significant SNP, 9_19840980, was associated with the RBC trait (*p* = 1.12e-10) (Figure 2). By means of genetic mapping and functional annotation, we identified five potential genes that were directly or indirectly related to high-altitude hypoxia adaptation (Table 2). Most of the SNPs were associated with MCH traits. Fourteen significant SNPs were associated with RBC and were located on Oar1, 2, 4, 8, 9, 13, 15, 17, and 21. Four SNPs were located within genes and others were located from 1,461 to 229,683 bp from the nearest gene. Two significant SNPs were associated with the HGB trait, one (17_53452406) of them was located within *CAMKK2* (calcium/calmodulin-dependent protein kinase kinase 2), and the other was located on Oar18. Only one significant SNP was associated with the HCT trait; it was located 10,803 bp away from LRP1B (LDL receptor-related protein 1B). Twenty-four significant SNPs were associated with MCH and were distributed on Oar1, 2, 3, 4, 7, 8, 9, 10, 11, 12, 13, 15, 17, 21, and 22. Eleven SNPs were located within genes, and others were located from 1,461 to 635,535 bp from the nearest gene. Six significant SNPs were associated with MCHC, three of these were located within genes including *VPS13D* (vacuolar protein sorting 13 homolog D) on Oar12, OTOA (otoancorin) on Oar24, and RSU1 (Ras suppressor protein 1) on Oar13, and the others were located from 67,432 to 496,548 bp from the nearest gene. Twenty significant SNPs were associated with the RWD_CV trait, these were distributed on Oar1, 2, 3, 4, 5, 8, 9, 11, 14, 15, 17, and 21, 12 of them were located within genes, and the others were located from 1,461 to 207,033 bp from the nearest gene.

**TABLE 2:** Associated SNPs and nearby potential genes for erythrocyte traits.

**Trait ^1^**	**SNP name**	**Oar ^2^**	**Position (bp) ^3^**	***P*-value**	**Nearest gene ^4^**	
					**Name**	**Distance (bp)**
RBC	1_29018708	1	29018708	1.48e-06	*DHCR24*	Within
MCH	10_80901397	10	80901397	2.86e-08	*EFNB2*	635,535 bp
MCH	17_54731492	17	54731492	1.28e-07	*SH2B3*	Within
MCH	1_29018708	1	29018708	1.45e-07	*DHCR24*	Within
MCH	13_798029	13	798029	1.28e-06	*PLCB1*	Within
RWD_CV	1_29018708	1	29018708	2.30e-07	*DHCR24*	Within
RWD_CV	17_54731492	17	54731492	2.75e-07	*SH2B3*	Within
RWD_CV	5_92277630	5	92277630	3.67e-08	*SPATA9*	Within
RWD_CV	5_92265355	5	92265355	1.82e-06	*SPATA9*	Within
RWD_CV	5_92276610	5	92276610	1.82e-06	*SPATA9*	Within
RWD_CV	5_92256711	5	92256711	1.91e-06	*SPATA9*	Within

*^1^ HCT, hematocrit; MCH, mean corpuscular hemoglobin; MCHC, mean corpuscular hemoglobin concentration; RWD_CV, RBC volume distribution width coefficient of variation. ^2^ The *Ovis aries* chromosome number of each significant SNP. ^3^ The positions of each significant SNP associated with erythrocyte traits in *Ovis aries* Oar_4.0 assembly. ^4^ The nearest annotated gene to each significant SNP. The annotated gene database is from https://www.ncbi.nlm.nih.gov/assembly/GCF_000298735.2. SNP is designated as in a gene or as distance (bp) from a gene region in *Ovis aries* Oar_4.0 assembly.*

### Significant Haplotypes

**FIGURE 3:**
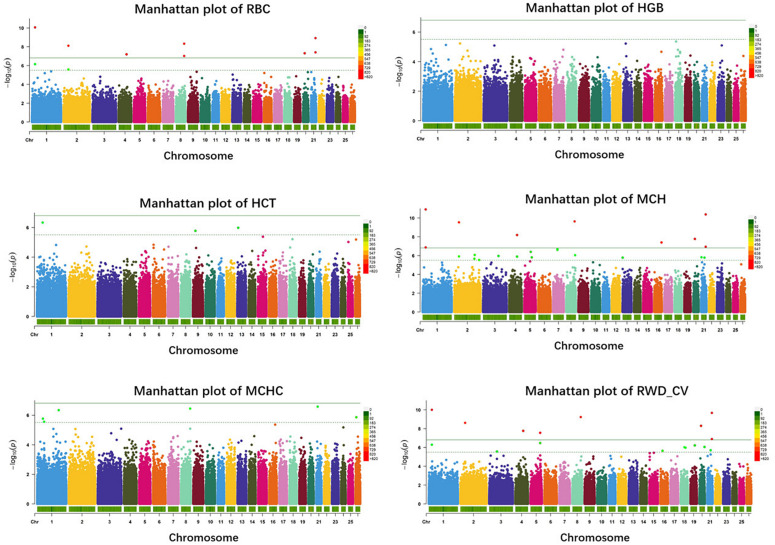
Manhattan plots of haplotype-based analysis for six erythrocyte traits. Chromosomes 1 to 26 are shown in different colors; the horizontal dotted green line and solid line indicate the suggestive and genome-wide significance level, respectively. Haplotypes surpassing the genome-wide threshold are highlighted in red, and haplotypes reaching the suggestive threshold are highlighted in green. The strips below each chromosome indicate the density of the markers. The six traits were: red blood cell count (RBC), hemoglobin (HGB), hematocrit (HCT), mean corpuscular hemoglobin (MCH), mean corpuscular hemoglobin concentration (MCHC), and RBC volume distribution width coefficient of variation (RWD_CV).

In haplotype analysis, we detected 32 significant haplotypes, including 24 suggested and eight genome-wide significant haplotypes. They were associated with five erythrocyte traits: 10 for RBC, 3 for HCT, 23 for MCH, 6 for MCHC, and 17 for RWD_CV, similarly to single-marker analysis, some of which were pleiotropic (haplotypes H1094_AA were associated with both RBC and MCH). These haplotypes were distributed on 15 autosomes, including Oar1, 2, 3, 4, 5, 7, 8, 9, 13, 16, 18, 19, 20, 21, and 26 (Figure 3). Of these 32 haplotypes, 13 were within or close to genes and the others were located from 1,668 to 1,176,920 bp away from the nearest genes (Supplementary Table S2). The most significant haplotype was associated with the RWD_CV trait (*p* = 1.21e-11) (Figure 3). By gene finding and functional annotation, we identified four potential genes that were directly or indirectly related to high-altitude hypoxia adaptation (Table 3). Most of the haplotypes were associated with MCH traits. Ten significant haplotypes were associated with RBC. Three significant haplotypes were associated with the HCT trait, which were located on Oar1, 9, and 13. Twenty-three significant haplotypes were associated with MCH and were distributed on Oar1, 2, 3, 4, 5, 7, 8, 13, 16, 20, and 21. Eight haplotypes were located close to or within genes, and others were located from 1,688 to 1,176,920 bp away from the nearest genes. Six significant haplotypes were associated with MCHC, two of them were located close to or within genes, and the others were located from 36,656 to 496,548 bp away from the nearest genes. Seventeen significant haplotypes were associated with the RWD_CV trait, 12 were distributed within genes, and the others were located from 6,529 to 229,076 bp away from the nearest genes.

**TABLE 3:** Associated haplotype blocks and nearby potential genes for erythrocyte traits.

**Trait ^1^**	**NSNP ^3^**	**Oar ^4^**	**Start ^5^**	**End ^5^**	**SNP1 ^6^**	**SNP2 ^6^**	***P*-value**	**Nearest gene ^7^**	
								**Name**	**Distance (bp)**
RBC	2	1	29018708	29023326	1_29018708	1_29023326	8.77e-11	*DHCR24*	Within
RBC	2	1	29018708	29023326	1_29018708	1_29023326	7.00e-07	*DHCR24*	Within
MCH	2	1	29018708	29023326	1_29018708	1_29023326	1.21e-11	*DHCR24*	Within
MCH	2	1	29018708	29023326	1_29018708	1_29023326	1.33e-07	*DHCR24*	Within
MCH	3	21	31336799	31343543	21_31336799	21_31343543	1.64e-06	*FLI1*	Within
MCH	2	13	798029	800471	13_798029	13_800471	1.66 e-06	*PLCB1*	Within
MCH	3	21	31336799	31343543	21_31336799	21_31343543	1.97e-06	*FLI1*	Within
RWD_CV	2	5	92277625	92277630	5_92277625	5_92277630	2.75e-08	*SPATA9*	Within
MCH	2	1	29018708	29023326	1_29018708	1_29023326	9.93e-11	*DHCR24*	Within
MCH	2	1	29018708	29023326	1_29018708	1_29023326	5.17e-07	*DHCR24*	Within

*^1^ MCH, mean corpuscular hemoglobin; RWD_CV, red blood cell volume distribution width coefficient of variation; HCT, hematocrit. ^2^ The name of significant haplotypes for each erythrocyte trait. ^3^ Number of SNPs included in haplotype block. ^4^ The *Ovis aries* chromosome number of each significant haplotype. ^5^ The start and end positions of each significant haplotype block associated with erythrocyte traits in *Ovis aries* Oar_4.0 assembly. ^6^ The first and second SNPs in the haplotype block. ^7^ The nearest annotated gene to each haplotype block. The annotated gene database is from https://www.ncbi.nlm.nih.gov/assembly/GCF_000298735.2. SNP is designated as a gene or as distance (bp) from a gene region in *Ovis aries* Oar_4.0 assembly.*

## Discussion

In the present study, both single-marker and haplotype-based analysis identified significant associations between erythrocyte traits with comparable sheep genomic regions. The main reason for using GLM in haplotype-based association studies is that the process of obtaining the genetic relationship constructed by haplotypes is complex; elimination of rare haplotypes and low-frequency individuals followed by subsequent association studies could avoid adverse effects on significance testing effectively. The basic principle of single marker GWAS is to compare phenotypic differences in allelic groups, it is more concerned about the effect of each marker. Significant SNP may loss caused by low linkage disequilibrium (LD) and mutation between markers if the density of markers is not high enough (Zhang et al., 2014), while the haplotype-based GWAS could effectively solve this problem, it is due to the fact that haplotype analysis could utilize both recent and ancestral reorganization information (Druet and Georges, 2010). However, haplotype analysis generally has greater degrees of freedom (df) than single-marker analysis, resulting in reduced detection capabilities (Zhang et al., 2012), it is difficult to balance between df and LD. In addition, the distribution of LDs in the whole genome is inconsistent, i.e., LD is higher in some districts and lower in other districts. Therefore, we conducted two different approaches to capture more associated SNPs. The differences in the numbers of markers (439,398 SNPs and 325,062 haplotypes) result in different *P*-values for the two methods. Although most of the genes involved are the same, the *P*-value distributions in the Manhattan plots from different analysis methods are slightly different. We detected some associations only via the SNP analysis, while other associations were detected only via the haplotype-based analysis. The two different methods detected that some of the associations were different. Therefore, the overlap markers detected by two different methods could make the results more reliable, and the markers of non-overlap may represent new valuable associations detected by different methods. Our results show that the efficiency of the method depends on the type of genotype data. This is consistent with the findings of Xiaoming et al. (2019) and of Wu et al. (2014).

In this study, we discovered 42 significant SNPs via single-marker analyses; six erythrocyte traits were involved, including RBC, HGB, HCT, MCH, MCHC, and RWD_CV. We detected five potential genes (*DHCR24, EFNB2, SH2B3, PLCB1*, and *SPATA9*) that were directly or indirectly related to hypoxic adaptation based on gene functional annotations, positions, and reported expression variation. Heart ischemia is a significant threat to livestock living on the plateau, which is not conducive to oxygen intake and exchange. Marker 1_29018708 on Oar1, associated with three different traits including RBC, MCH, and RWD_CV, was located within *DHCR24* (24-dehydrocholesterol reductase). It has been reported that increased expression of this gene could activate phosphatidylinositol 3-kinase and induce *HO-1* (heme oxygenase-1) (Wu et al., 2012). *HO-1* induction prevents heart ischemia and reperfusion injury (Otterbein et al., 2003), while the normal function of the heart ensures blood circulation and oxygen transport, which is critical for livestock to survival in hypoxic conditions. *EFNB2* (ephrin B2) encodes a member of the ephrin (EPH) family, and the EPH receptors are the largest subfamily of receptor tyrosine kinases (*RTK*s), which have been implicated in mediating developmental events, especially in the nervous system and in erythropoiesis (Bennett et al., 1995; Suenobu et al., 2002). Duffy et al. (2010) pointed out that mesenchymal stem cells (MSCs) expressing *EFNB2* could promote cardiovascular formation in ischemic tissue; a very significant marker (10_80901397, *p* = 2.86e-08) found in our study was located 635,535 bp away from this gene. In addition, another gene, *SH2B3* (SH2B adaptor protein 3), closely related to hematopoietic function, was revealed in this study. Marker 17_54731492 on Oar17, associated with MCH and RWD_CV, was located within this gene that encodes a member of the *SH2B* adaptor family of proteins, which is an adaptor protein regulating the production of B cells (Li et al., 2000). Satoshi et al. found that *SH2B3* is expressed in early hematopoietic progenitor cells and that the hematopoietic capacity of hematopoietic stem cells (HSCs) increases significantly when the expression of SH2B decreases. They speculated that this gene plays a key role in HSC amplification and function (Takaki et al., 2000). All red blood cells are formed by differentiation of HSCs, and HSCs would self-renew throughout the life cycle of the body to continuously generate new blood cells (Till et al., 1964; Reya, 2003). Marker 13_798029 on Oar13, associated with MCH, was located within PLCB1 (phospholipase C beta 1). Several studies have proved that *PLCB1* participates in the body’s hematopoietic system and regulates the differentiation and production of red blood cells (Cocco et al., 2015). Follo et al. (2012) determined that *PLCB1* inhibited the differentiation process of red blood cell lines, while another isoenzyme, PI-PLC gamma, increased during the differentiation of red blood cells, which regulate the production of normal red blood cells. A total of four SNPs were located on Oar5 (Supplementary Table S1), associated with the RWD_CV trait, and were located within *SPATA9* (markers 5_92277630, 5_92265355, 5_92276610, and 5_92256711). Artigas et al. identified 16 new loci that affect lung function through genome-wide association and large-scale follow-up (46,411 individuals), including *SPATA9, TGFB2, C10orf11*, and *LRP1*; they found that SPATA9 was expressed in the lungs, airway smooth muscle, bronchial epithelial cells, and peripheral blood mononuclear cells. Moreover, they also found that this gene was associated with the forced expiratory volume in 1 s (FEV1) and the ratio of FEV1 to forced vital capacity (FVC) (Soler Artigas et al., 2011). Interestingly, a reduced ratio of FEV1 to FVC is used to define airway obstruction, and a reduced FEV1 is used to grade the severity of airway obstruction (Jakeways et al., 2003). These two parameters directly affect the absorption and utilization of oxygen by livestock in low-oxygen environments.

We identified 34 significant haplotypes associated with the five erythrocyte traits. By genetic mapping and functional annotation of these haplotypes, three important genes, including *DHCR24* on Oar1, *PLCB1* on Oar13, and *SPATA9* on Oar5, were overlapped with single-marker GWAS, which will not be discussed further in this section. In addition to the above three genes, we identified another important gene in the haplotype analysis, *FLI1* (Fli-1 proto-oncogene, ETS transcription factor). One significant haplotype block associated with MCH was found within this gene. *FLI1* is a member of the ETS transcription factor family, and analysis of the expression of members of this family in hematopoietic tissues, as well as studies of targeted mutations in ETS family members, suggest that they are critical for regulation of normal hematopoietic development (Graves and Petersen, 1998). Spyropoulos et al. (2000) demonstrated that hematopoiesis is severely impaired in *FLI1* mutant mice at mid-gestation, and they speculated that the loss of function of *FLI1* may lead to disruption of normal hematopoietic function in mice. Based on our results from single-marker GWAS and haplotype analysis, we speculate that the expression of these six genes may be related to high-altitude hypoxia adaptation, and we will continue to investigate the biological functions of the above potential genes.

## Conclusion

We identified 42 SNPs and 34 haplotypes, some of which were pleiotropic, such as 8_82239902, and were associated with both RBC and RWD_CV. Six genes were selected as potential for genes involved in high-altitude hypoxia adaptation based on functional annotations and gene mapping. In particular, *PLCB1* and *FLI1* are associated with the production of RBCs and normal hematopoietic function, respectively. The current results provide a valuable reference for studies of high-altitude hypoxia adaptation in domestic animals, such as the Alpine Merino sheep, living on high-altitude plateaus. Further research on these genes will be conducted, and the identified SNPs and haplotypes will be investigated in other populations and functionally verified to identify causal mutations.

## Data Availability Statement

The datasets presented in this study can be found in online repositories. The names of the repository/repositories and accession number(s) can be found below: https://figshare.com/articles/dataset/Phenotype_dataset_blood_498_txt/12072261, https://figshare.com/articles/dataset/Genotype_dataset_GWASQC_498_vcf/12072249.

## Ethics Statement

The animal study was reviewed and approved by the Animal Management and Ethics Committee of Lanzhou Institute of Animal Husbandry and Veterinary Medicine, Chinese Academy of Agricultural Sciences. Written informed consent was obtained from the owners for the participation of their animals in this study.

## Author Contributions

SZ and TG conceptualized the study. SZ and CY contributed to the data curation. SZ and TG contributed to the formal analysis. BY contributed to the funding acquisition. HZ and MH investigated the study. TG performed the methodology. YY and BY contributed to the project administration. TW and FL contributed to the resources. JL and CY contributed to the software. HZ contributed to the validation. GQ visualized the study. SZ and TG wrote the original draft. YY and BY wrote, reviewed and edited the manuscript. All authors contributed to the article and approved the submitted version.

## Conflict of Interest

The authors declare that the research was conducted in the absence of any commercial or financial relationships that could be construed as a potential conflict of interest.

## Footnotes

1 https://cran.r-project.org/web/packages/CMplot/index.html

## Supplementary Material

FIGURE S1 Population structure map drawn from the first three principal components.

TABLE S1 All associated SNPs and nearby genes for erythrocyte traits.

TABLE S2 All associated haplotype blocks and nearby genes for erythrocyte traits.
