# Supplementary figures and images for: Genome-Wide Association Study Using Individual Single-Nucleotide Polymorphisms and Haplotypes for Erythrocyte Traits in Alpine Merino Sheep
Source: Front Genet. 2020 Jul 31;11:848. doi: 10.3389/fgene.2020.00848 (PMC7411260; doi:10.3389/fgene.2020.00848)

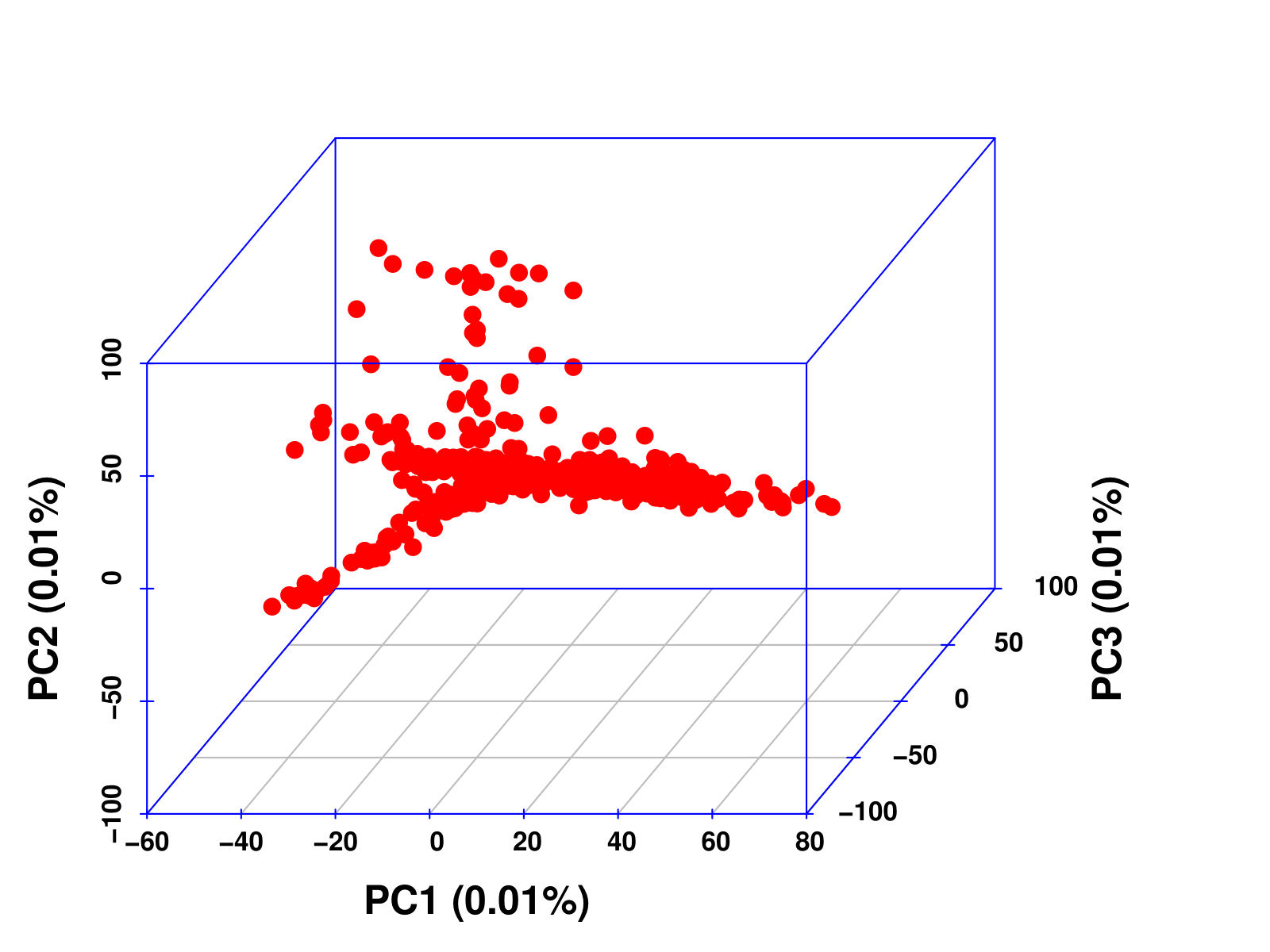

Supplement: FIGURE S1 — Population structure map drawn from the first three principal components. [file Image_1.tiff]
